# Preparation, Statistical Optimization and *In-vitro* Characterization of a Dry Powder Inhaler (DPI) Containing Solid Lipid Nanoparticles Encapsulating Amphotericin B: Ion Paired Complexes with Distearoyl Phosphatidylglycerol

**DOI:** 10.22037/ijpr.2019.15208.12963

**Published:** 2020

**Authors:** Ehsan Mehrabani Yeganeh, Hossein Bagheri, Reza Mahjub

**Affiliations:** *Department of Pharmaceutics, School of Pharmacy, Hamadan University of Medical Sciences, Hamadan, Iran.*

**Keywords:** Amphotericin B, Ion paired complexation, Distearoyl phosphatidylglycerol (DSPG), Solid lipid nanoparticle (SLN), Dry powder inhaler (DPI), Lyophilization technique

## Abstract

The aim of this study was to prepare dry powder inhalers (DPIs) containing amphotericin B-loaded solid lipid nanoparticles (AMB-SLNs) as an alternative approach for prevention of pulmonary aspergillosis. For solubilizing AMB in small amounts of organic solvents ion paired complexes were firstly formed by establishing electrostatic interaction between AMB and distearoyl phosphatidylglycerol (DSPG). The SLN formulations containing AMB-DSPG complexes were prepared using glycerol monostearate (GMS) as the lipid matrix and soybean lecithin and tween 80 as the surfactants by solvent emulsification-evaporation technique. The nanoparticles were optimized through a fractional factorial design. DPIs were prepared by lyophilization technique using lactose as the inhalational carrier and then after, the formulations were evaluated in terms of aerodynamic particle size distribution using an Andersen cascade impactor. The morphology of the particles was examined using scanning electron microscopy (SEM) and *in-vitro *drug release profiles were evaluated. Following the statistical results, the particle size, Poly dispersity index (PdI), zeta potential, entrapment efficiency (EE%), and drug loading (DL%) of the optimized SLNs were 187.04 ± 11.97 nm, 0.188 ± 0.028, -30.16 ± 1.6 mV, 89.3 ± 3.47 % and 2.76 ± 0.32 %, respectively. Formulation containing 10% w/v of lactose with the calculated fine particle fraction value as 72.57 ± 4.33% exhibited the appropriate aerodynamic characteristics for pulmonary drug delivery. SEM images revealed de-agglomerated particles. *In-vitro *release studies showed sustained release of AMB from the carriers and the release kinetics were best fitted to the first order kinetic model.

## Introduction

In recent years, pulmonary fungal infections have been classified as a serious global health problem and a major cause of mortality and morbidity in immunocompromised patients (1). Among these infections, invasive pulmonary aspergillosis (IPA) is known as the most common fungal pulmonary infection in susceptible individuals such as patients, suffering from severe neutropenia, allogeneic hematopoietic stem cell or solid organ transplant recipients as well as those who are undergoing intensive anti-cancer chemotherapy and patients receiving prolonged immunosuppressive therapy especially with corticosteroids (2). Unfortunately, common antifungal medications show low efficiency while exhibit toxic effects to the host. During conventional systemic administration, drug dilution and degradation in the blood along with off-target drug distribution are some aspects of limitations for delivering intended dose to the infected sites. Salma *et al.* reported development of acute immune intravascular hemolysis due to a degradation product of Amphotericn (3). Moreover, Wang *et al.* suggested that the temperature -induced degradation of Amphotercin B in plasma can occur due to auto-oxidation of the compound at polyene moieties by free radical mechanism (4). To overcome these problems and achieving an effective treatment, large dose of antifungals must be given and consequently, the risk of eventual adverse effects can be increased (5).

Pulmonary drug delivery as an alternative non-invasive approach has received more attention. In this strategy, the intended drug can be delivered directly to the disease site and accordingly leads to decreased systemic absorption and therefore reduces the risk of adverse effects. Furthermore, this route of administration represents high potential for treating local infections in pulmonary site (6, 7). In recent years, application of dry powder inhalers (DPIs) has been emerged as an efficient inhalational drug delivery system. DPIs, among other pulmonary drug delivery systems, have some advantages including greater chemical stability of the active compound and broader dosing options. Moreover, in contrast with metered dose inhalers (MDIs), the drug delivery system is free of propellant and little or no patient coordination is required (8).

Amphotericin B (AMB) is a polyene antifungal with a broad spectrum of activity against different fungi species. In spite of introduction of new agents such as voriconazole and itraconazole, AMB is still designated as the first choice in IPA treatment and prophylaxis for decades (9, 10). Notwithstanding the clinical efficiency of conventional AMB (amphotericin B deoxycholate, Fungizone^®^) as a prophylactic agent, its application is limited by frequent and severe side effects including nephrotoxicity, fever, chill, nausea, vomiting, and anemia (11, 12).

Solid lipid nanoparticles (SLNs) have been introduced recently as an alternative carrier system to the other colloidal carriers such as emulsions, liposomes, and polymeric nanoparticles (13). Special characteristics of SLNs such as small size, large surface area, high drug loading, the interaction of phases at the interfaces along with good biocompatibility, low toxicity, controlled release profile, drug targeting to specific organs and high stability make them suitable for administration through different routes including parenteral, dermal, pulmonary, and topical approaches (14). Moreover, inhalational drug delivery system provides a high drug concentration in the respiratory tract accompanied by decreased side effects. Therefore, aerosolized antifungal therapy represents an efficient alternative approach for prevention of IPA (15).

**Figure 1:**
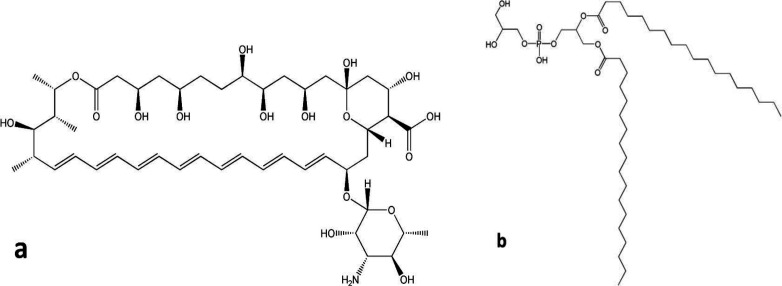
Chemical structure of (a) amphotericin B; (b) DSPG, (ChemDraw Ultra v. 7.0 software).

As shown in Figure 1a, AMB as a polyene antifungal contains a large lactone ring including seven double bonds in an aliphatic chain while the other side of the lactone ring is occupied by numerous hydroxyl groups. Furthermore, the polyene represents ionizable groups, such as carboxylic acid and amine groups in the chemical structure. Structurally, double bonds of aliphatic chain form hydrophobic moieties while polyol and ionizable groups were considered as the hydrophilic part of the molecule. Therefore, AMB molecule exhibits amphiphilic characteristics with low solubility in aqueous and most organic solvents (5, 16). According to this sparing solubility, AMB shows nearly no oral absorption and its gastrointestinal bioavailability is limited. By considering this fact and in order to achieve effective antifungal concentration, AMB must be administered parenterally (17). Preparation of AMB-distearoyl phosphatidylglycerol (DSPG) electrostatic complexes was considered as a practical way for solubilizing AMB in small amount of organic solvents. It was reported that formation of the ion paired complex of DSPG-AMB can provide a new and efficient strategy for improving the poor solubility of amphotericin in organic solvents (5). Accordingly, the strongly associated complex is highly soluble in small amounts of organic solvents such as methanol and dichloromethane which were used in this study for preparation of nano-carriers. The chemical structure of DSPG is illustrated in Figure 1b.

In this study, we developed a new DPI formulation based on preparation of solid lipid nanoparticles entrapping AMB-DSPG complexes as the alternative way for pulmonary delivery of AMB. 

## Experimental

*Materials*

AMB, DSPG and glycerol monostearate (GMS), dimethyl sulfoxide (DMSO) and dialyzing tube with a molecular cutoff of 12,000 Da (D0405) were purchased from Sigma™ (St. Louis, MO, USA). Lactose monohydrate, Soybean lecithin, polyethylene glycol 400, tween 80 and sucrose were supplied by Samchun™ (Seoul, Korea). Sodium chloride, potassium chloride, disodium hydrogen phosphate, potassium dihydrogen phosphate, and HPLC grade solvents including methanol, acetonitrile and acetic acid were obtained from Merck™ (Darmstadt, Germany). Analytical grade water was provided using Milipore^®^ water purification system. All other ingredients were of pharmaceutical grade and were used as received.

*Preparation of the AMB-DSPG ion paired complexes*

Ion paired complexes of AMB-DSPG were prepared according to previous reports, with minor modifications (5). Briefly, DSPG (8 mg) was dissolved in equal mixture of dichloromethane and methanol (5 mL) at 65 ºC to yield a clear solution and then acidified using hydrochloric acid (2.5 N) to adjust the pH at 3.0. Then after, 10 mg of AMB was firstly suspended in equal mixture of dichloromethane and methanol (5 mL) and then added to the previously prepared acidified solution. Afterwards, the mixture was heated at 65 ºC for 10 min while kept stirring at 500 rpm. During the ion pair complexation, firstly the phosphate group of DSPG molecule tends to accept a proton in the acidified solution and consequently forms a relatively neutral molecule. After mixing AMB with the previously prepared solution of DSPG, carboxyl group of AMB would tend to accept the proton of the phosphate group related to DSPG and accordingly positive charge of AMB would be established. On the other hand, DSPG by donating proton forms a negatively charged molecule. Therefore, an electrostatic interaction between positively charged AMB and negatively charged DSPG would become established.

*Preparation of the SLNs*

The SLNs were prepared using solvent emulsification-evaporation method (18, 19). Briefly, GMS and soybean lecithin were dissolved in dichloromethane as the organic solvent by stirring at ambient temperature. Then, 2 mL of AMB-DSPG ion paired complexes was added to the previously prepared lipid phase and kept stirring for a period of five minutes. The prepared organic phase was added dropwise to a previously heated aqueous solution containing various amounts of tween 80 and PEG 400 at 50 °C while stirring using a silent crusher homogenizer at 5,000 rpm for predetermined time period referred as emulsification time for evaporation of the organic solvents. The final dispersion was immediately transferred into the ice bath at 0 °C under same condition of stirring for predetermined time period designated as cooling time. The SLNs were obtained by centrifugation of the freshly prepared colloidal suspension at 20,000 rpm for 30 min at 4 °C using Beckman-Coulter Optima™ XPN-100 ultracentrifuge. Supernatant was separated and collected for subsequent analysis using previously reported HPLC method while the settled down SLNs were collected, re-suspended in distilled water and used for further experiments (20). Optimization of the nanoparticles was performed using fractional factorial design and eventually the optimized nanoparticles were lyophilized using sucrose 5% (w/v) as the cryoprotectant by Operon® (FDB55039, South Korea). The effects of the lyophilization process on physicochemical characteristics of the nanoparticles were also studied by comparing the characteristics of the nanoparticles before and after lyophilization using two sample independent t-test statistical analysis performed by SPSS® software (V16.0.0).

*Characterization of the nanoparticles*

The mean particle size (Z-average) and polydispersity index (PdI) of the SLNs were determined by dynamic light scattering (DLS) using a Malvern® zetasizer- nanosizer (Malvern® instruments, United Kingdom). Zeta potential of the nanoparticles was evaluated by the same instrument using electrophoretic mobility of nanoparticles based on Smoluchowski’s equation. All measurements were performed at ambient temperature of 25 °C.

The percentage of entrapment efficiency (EE%) and drug loading (DL%) was indirectly calculated by the following Equations (21): 

EE% = ((Total drug content – drug content in the supernatant)/Total drug content)) × 100

 (Equation 1)

DL% = ((Total drug content – drug content in the supernatant)/Total weight of nanoparticles) × 100 

(Equation 2)

*Experimental design studies*

According to literature, various methods have been used for statistical optimization in preparation of nanoparticles (22). In this study, preparation of the SLNs was statistically optimized using a fractional factorial design consists of five independent factors designated as concentration ratio of GMS/lecithin (A), quantities of PEG 400 (B) and tween 80 (C) as well as emulsification time (D) and cooling time (E). The two dependent variables were defined as particle size (Y1) and polydispersity index (PdI) (Y2). The appropriate range and constrains of independent variables (factors) were determined using preliminary studies and are summarized in Table 1. The factorial design experiments were carried out in 11 sets with different values of independent variables. According to preliminary studies, the amounts of AMB-DSPG complex and soybean lecithin were kept constant at 2 mL and 50 mg, respectively, whereas, the quantities of GMS, tween 80, PEG 400, emulsification time and cooling time were varied. Different experimental runs with various compositions as F1 to F11 were designed as shown in Table 2. All the measurements were performed in triplicate. All results were analyzed statistically using Design-Expert® Software (V.7.0.0, Stat-Ease, Inc. Minneapolis, USA). The formulations were experimentally prepared as designed by the software and the significance of interaction between variables was statistically evaluated using one-way analysis of variance (ANOVA). 

In model fitting analysis, the responses, namely particle size (Y1) and pdI (Y2), of all the formulations were treated by the software and for each response, best fitting mathematical model was determined on the basis of comparison of several statistical parameters including coefficient of variation (CV), R-Squared (R2), adjusted R-squared (adjusted R2), and adequate precision provided by Design-Expert® software. The level of significance was considered at *p *< 0.05. The co-efficient of each significant effect was further used to develop a reduced equation by step-wise multiple regression analysis. Some of the interactions between independent variables were visually explained by using 3-D response surface plots.

*Model validation*

In order to validate the experimental model and evaluation of the prediction errors, the optimized formulation suggested by the software was prepared experimentally in five times and characterized in terms of particle size, polydispersity index (PdI), zeta potential (mV), EE% and DL% using Malvern zetasizer- nanosizer and HPLC as previously discussed.

*Development of DPI formulation containing SLNs*

Dry powders containing AMB-SLNs were obtained by lyophilization technique (23, 24). Briefly, an aqueous solution of lactose as an inhalational carrier was added to the previously optimized colloidal suspension of nanoparticles. In order to achieve a suitable dry powder characteristics, various concentrations of lactose including 1%, 5%, 10%, 15%, and 20% w/v were provided in AMB-SLNs re-constituted colloidal suspensions. The prepared formulations were lyophilized using an Operon™ freeze dryer (FDB 5503, Korea). Following lyophilization, the powders were sieved through a #200 mesh sieve and their related aerodynamic size distribution were analyzed using an 8-stage Anderson cascade impactor (Westech^®^ scientific instrument, Upper Stondon, United Kingdom). Fine particle fraction (FPF) defined as the percentage of particles with diameter between 1-5 µm was determined by calculating the amount of drug deposited under stage 4 (cut off point of 5 µm) and above stage 7 (cut off point of 0.93 µm) divided to the total amount of the drug.

*Determination of the morphology of the particles*

The lyophilized nanoparticles were examined by scanning electron microscopy (SEM) performed by a JEOL JSM-840 SEM. The samples for SEM analysis were prepared by placing drops of the fresh nanoparticles dispersion on carbon-coated SEM copper grids covered with a glass lamella. The mixtures were allowed to dry for some minutes and then gold coated under vacuum and examined in terms of related size and shapes. Morphology of the DPI formulation was also carried out by scanning electron microscopy equipped with an image analyzer using the same treatment. 

*In-vitro release studies*

The optimized and experimentally developed formulation of SLNs was subjected to *in-vitro *release studies according to previous reports (25, 26). Proper volume of AMB-SLNs colloidal suspension equivalent to 2 mg of AMB was placed in dialysis bag (MWCO 12 kDa) and was subsequently immersed in 100 mL of the preheated PBS (pH 7.4) and incubated at 37 ± 2 °C while stirring at 100 rpm using Heidolph™ shaker incubator (Heidolph™ Unimax 1010/Incubator 1000, Germany). The volume of the release medium was adjusted in a manner that sink condition was ascertained. At predetermined time intervals, 1 mL of the release medium was withdrawn and was replaced by the equal volume of freshly prepared pre-heated buffer. Drug concentration in the samples was determined using HPLC (Shimadzu, LC-20AD) at 406 nm. All experiments were carried out in triplicates. 

Release studies of the DPI formulations were also performed as the same method applied for the nanoparticles by using suitable amount of the DPI formulation equivalent to 2 mg of AMB which was dispersed in 5 mL of distilled water and sonicated for 2 min using Bandelin™ ultrasonic bath (DT 102H, Germany) and then placed in the dialysis bag and immersed in PBS. 

In order to evaluate the release kinetic of nanoparticles and DPIs, release data were fitted to various release models such as zero order, first order, Higuchi, Korsmeyer-peppas and Hixson Crowell and their appropriate correlation coefficients were investigated using SigmaPlot® software (version 10.0.0.54).

*Statistical analysis*

In this study all experiments were performed in triplicate except otherwise stated which were carried out experimentally in five times. Comparison of two groups of data was performed using two sample independent *t*-test by SPSS® software (V.16.0). Factional factorial design and model fitting were accomplished using Design-Expert® software (V.7.0.0). The significance level was set as 0.05.

## Results and Discussion

*Preparation and characterization of the SLNs*

Establishment of electrostatic interactions between amphotericin and DSPG provides an approach to overcome the poor solubility of polyenes in organic solvents. In this study, suspended dispersion of AMB in organic solvents (*i.e. *combination of methanol and dichloromethane) was completely dissolved in the presence of acidified DSPG and resulted in formation of an orange transparent solution without presence of any colloidal or suspended particles indicating formation of AMB-DSPG complex. The concentration of AMB-DSPG complex was evaluated by HPLC and reported as 960 ± 14.21 µg mL^-1^ while the concentration of equal amount of AMB dissolved in the same organic solvents in the absence of DSPG was reported as 345 ± 5.76 µg mL^-1^ indicating significant role of complexation in enhancing the solubility of AMB in the organic solvents. 

In this study, fractional factorial design was employed for preparation of the SLNs to evaluate all the main effective and possible binary interactions to determine which independent variables and interactions have significant inﬂuence on the defined responses. In this case, 11 formulations of AMB-loaded SLNs were prepared by solvent emulsification-evaporation method, using a 25- fractional factorial design accompanying with three center points. The values of independent variables and the related experimental data in suggested formulations (*i.e.* F1-F11) are summarized in Table 2.

In this study, analysis of responses performed using design-expert software showed that dependent variables including particle size (Y1) and pdI (Y2) were both fitted to 2-factorial interaction (2-FI) model with the model *p*-value of 0.0161 and 0.0023, respectively. The values of R2, adjusted R2, Adeq precision, SD and CV% are summarized in Table 3.

*Size of the nanoparticles*

Particles with mean diameter ranging from 176.7 ± 5.09 nm to 393.6 ± 47.5 nm were obtained in various suggested runs as shown in Table 2. Statistical analysis performed by Design-Expert® based on fractional factorial design was applied to establish the best significant fitted model for prediction of the size of particles. The characteristics of fitted model are summarized in Table 3. The analysis of variance for data revealed that the linear coefficients of the all independent factors, except factor E, and interaction coefficients of BC and BE were significant (*p *< 0.05). The coefficients of significant variables on particle size (Y1) have been shown in Equation 3 as follows:

 Y1 = +219.46 + 21.84A - 18.76B + 32.44C - 38.24D - 15.94BC - 34.66BE 

 (Equation 3)

Where: 

Y1: Particle size;

A: Coefficient of GMS/Lecithin

 concentration ratio; 

B: Coefficient of PEG 400 concentration (%); 

C: Coefficient of Tween 80 concentration (%); 

D: Coefficient of emulsifying time (h); 

E: Coefficient of cooling time (h); 

BC: Interaction coefficient of B and C; 

BE: Interaction coefficient of B and E; 

As shown in the equation, both coefficients of A and C showed a positive effect on the size of nanoparticles (Y1). It means that particle size would be expected to be increased with increasing either concentration ratio of GMS/Lecithin (A) or concentration of Tween 80 (C). GMS, as the central core, forms the lipid matrix of the SLNs. Therefore, it is sensible that an increased particle size can be observed with higher concentration ratios of GMS/lecithin (27). This phenomenon was in well accordance with previous studies which showed the dependency of the size of SLNs on the concentration of GMS as the lipid matrix and it was previously reported that increased amount of GMS caused an increase in particle size which can be explained in terms of tendency of the lipid to coalesce at high concentration. Based on Stoke’s law, the difference in density between internal and external phase can interpret this behavior (28). Moreover, the study of Mehnert and Mader demonstrated that particle size of SLNs would be increased by increasing the lipid content of nanoparticles (29). This can be due to a decrease in homogenization efficiency by increasing the viscosity of inner phase followed by increasing the lipid concentration (27, 30).

In this study, tween 80 was selected as the surfactant and as indicated in Equation 3, the positive sign of the related coefficient demonstrates that increased level of tween 80 can lead to particle size increasing of the prepared SLNs. This can be justified by considering that the high concentration of surfactants causes an increase in viscosity of aqueous phase which in turn, reduces the homogenization efficiency and consequently decreases the tendency for particles breakdown (31). Moreover, high concentrations can promote aggregation of the surfactant molecules at the surface of particles along with establishment of loops and tails which consequently leads to bridging between the nanoparticles and promotes aggregation. The studies performed by Tiyaboonchai *et al.* revealed an increase in particle size of SLNs followed by increasing the concentration of surfactant (32). In some studies it was suggested that in increased concentration of surfactants, establishment of strong intermolecular interaction via hydrogen bonds between surfaces of prepared SLNs can lead to particle size increasing and tendency of aggregation in nanoparticles (33).

As confirmed by Equation 3, concentration of PEG 400 (B) as well as emulsification time (D) had negative effects on the particle size (Y1). It means that the particle size would be expected to be decreased with increasing either concentration of PEG 400 or emulsification time. Polyethylene glycol (PEG), through its characteristics, can act as an hydrophilic shield in SLNs and other drug carriers and consequently reduces the surface tension of SLNs and can lead to a decrease in particle size (34). Furthermore, PEG 400 can provide favorable conditions to establish a more stable and dispersed formulation of nanoparticles (22). In accordance with this observation, Liu *et al.* (33) have suggested PEG 400 as the ideal co-surfactant to prepare diclofenac-loaded SLNs with a relative smaller particle size. Negative sign of the coefficient of factor D (*i.e.* emulsification time) demonstrates that increased duration of homogenization cycle can lead to decrease in particle size, since generated shear force can have more available time for particles breakdown (31).

**Figure 2:**
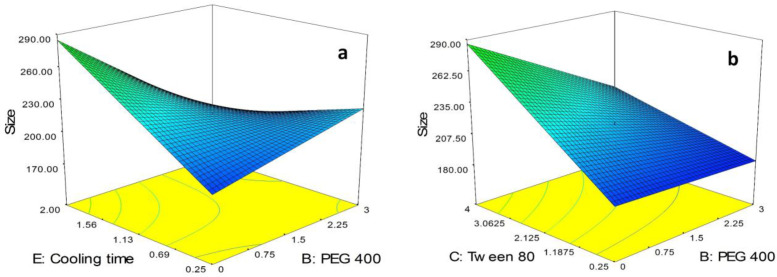
3D plots of effective binary interactions on particle size, (a) BE interaction, (b) BC interaction

As can be seen in Equation 3, BC and BE as the binary interactions showed a significant effect on particle size of the SLNs. In order to study the interaction patterns between variables, 3D response surface curves were plotted by model prediction of the particle size (Y_1_) at different levels of the effective two variables while keeping the other variables at their center levels (Figure 2). As shown in Figure 2a, although in lowest value for cooling time (*i.e.* 0.25 h), the size of particles was slightly increased followed by increasing the concentration of PEG 400 from 0.0% to 3.0%, in highest value for cooling time (*i.e.* 2.0 h) a sharp decrease in the size of particles was observed followed by increasing the concentration of PEG 400. Moreover, it is obvious from the figure that in the absence of PEG 400, size of the SLNs was sharply increased by increasing the cooling time from 0.25 h to 2.0 h while in highest concentration of PEG 400, a decrease in the size of the particles was observed due to increase in cooling time. 

As shown in Figure 2b, in lowest concentration of tween 80 (*i.e.* 0.25%), increasing the concentration of PEG 400 from 0.0% to 3.0% had no significant effect on the size of the particles while in highest concentration of tween 80 (*i.e.* 4.0%), a sharp decrease in the size of the SLNs was observed followed by increasing the concentration of PEG 400. On the other hand, as can be seen in the figure, in absence of PEG 400, the size of particles was sharply increased by increasing the concentration of tween 80 from 0.25% to 4.0%. The same trend with slower rate was observed for increasing the size of particles due to increase in concentration of tween 80 in the presence of high concentration of PEG 400 (*i.e.* 3.0%). 

*PdI of the nanoparticles*

As shown in Table 2, the experimentally observed PdI is ranged from 0.17 ± 0.015 to 0.491 ± 0.03. Homogeneity of nanosuspensions becomes higher as the PdI approach to zero (30). Statistical analysis performed by Design-Expert® based on fractional factorial design was applied to establish the best significant fitted model for prediction of the PdI. The characteristics of the best fitted model are summarized in Table 3. Analysis of variance for data revealed the linear coefficients of the all independent factors except factor C, and interaction coefficients of B.C and B.E were significant (*p* < 0.05). The coefficients of the significant variables on pdI (Y2) have been shown in Equation 4 as follows:

Y_2 _= +0.30 - 0.055 A - 0.022 B - 0.027D + 0.033E + 0.022BC - 0.075BE 

 (Equation 4)

Where: 

Y_2_: PdI of Particles; 

A: Coefficient of GMS/ Lecithin

 concentration ratio; 

B: Coefficient of PEG 400 concentration (%); 

C: Coefficient of Tween 80 concentration (%); 

D: Coefficient of emulsifying time (h); 

E: Coefficient of cooling time (h); 

BC: Interaction coefficient of B and C; 

BE: Interaction coefficient of B and E;

As confirmed by Equation 4, three factors including concentration ratio of GMS/Lecithin (A), concentration of PEG 400 (B), and emulsification time (D) had negative effects on PdI of the nanoparticles (Y2). It means that PdI would be expected to be decreased by increasing either factor A, B or D while cooling time as factor E revealed a positive effect on PdI of the particles.

**Figure 3:**
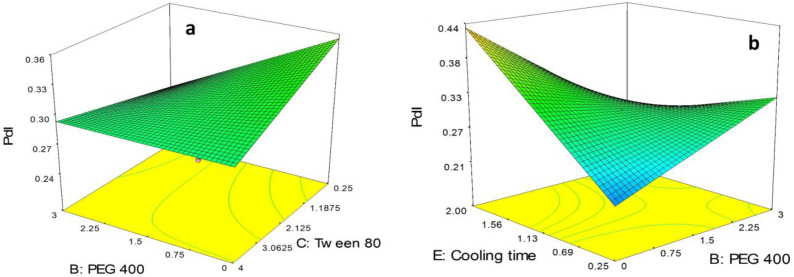
3D plots of effective binary interactions on pdI, (a) BC interaction, (b) BE interaction

As can be seen in Equation 4, BC and BE as the binary interactions showed a significant effect on PdI. In order to study the interaction patterns between variables, 3-D response surface curves were plotted by model prediction of the PdI at different levels of the effective two variables while keeping the other variables at their center levels. The 3-D response surface plots of observed PdI are provided in Figure 3.

As shown in Figure 3a, although in the absence of PEG 400, PdI of the nanoparticles was slightly decreased by increasing the concentration of tween 80 from 0.25% to 4.0%, in high concentration of tween 80 (*i.e.* 4%) the alteration in the PdI due to increase in concentration of PEG 400 from 0.0% to 3.0% was not significant. Moreover, it is obvious in the figure that in low concentration of tween 80 (*i.e.* 0.25%) the polydispersity of the particles was sharply decreased followed by increasing the concentration of PEG 400 from 0.0% to 3.0%. 

As illustrated in Figure 3b, in the absence of PEG 400, PdI of the nanoparticles was sharply increased by increasing in cooling time from 0.25 h to 4.0 h. On the other hand, in highest concentration of PEG 400 (*i.e.* 3.0%) a slight decrease in PdI of the particles was observed. Moreover, in lowest value for cooling time (*i.e.* 0.25 h), PdI of the particles was observed to increase by increasing the concentration of PEG 400 from 0.0% to 3.0% while in highest values for cooling time (*i.e.* 2.0 h) a sharp decrease in PdI of the SLNs was observed followed by increasing the concentration of PEG 400. 

*Optimization and model validation*

Optimization of the physicochemical characteristics of the SLNs was carried out according to statistical analysis of the experimentally obtained data using fractional factorial design. The criteria for dependent variables (*i.e.* size and PdI) were previously stated as the constrains in Table 1. The optimized conditions for preparation of the SLNs which were predicted by the Design-Expert® software using modeling and regression analysis, are shown in Table 4. To determine the model validation and calculation of the appropriate prediction error, the suggested optimized formulation was prepared and characterized experimentally (n = 5). The observed responses and value of predicated errors are indicated in Table 5. As shown in the table, the calculated prediction errors were well below 10% for all items demonstrating the proper predictability, efficiency and adequacy of the fitted models. EE%, DL% and zeta potential are critical parameters for evaluation of physicochemical characteristics of submicron systems. Accordingly, EE% of the optimized SLNs formulation was calculated and determined as high as 89.3 ± 3.47% which demonstrates that the AMB-DSPG complexes can be successfully loaded into the nanostructures. Drug loading (DL%) is ordinarily defined in percent related to the lipid phase that was determined to be 2.76 ± 0.32 (Table 5). Zeta potential of the particles considered as an indicator for prediction of the stability of the colloidal dispersion (35). Accordingly, particle aggregation is less likely to occur in high zeta potentials (either positive of negative) due to high electrostatic repulsion forces between particles (36). Therefore, nanoparticles with zeta potential values greater than +20 mV or less than -20 mV exhibit high degrees of stability (37). As shown in Table 5, zeta potential of the optimized SLNs was determined as -30.16 ± 1.6 mV which can ensure proper stability for the AMB loaded SLNs. According to the previous studies, the negative charge of zeta potential is related to the lipids that incorporate into the SLNs structure (38, 39). In this study, surface accumulation of GMS as the main lipid in the structure of the SLNs resulted in high negative zeta potential of the nanoparticles. 

*Lyophilization of the nanoparticles*

**Figure 4:**
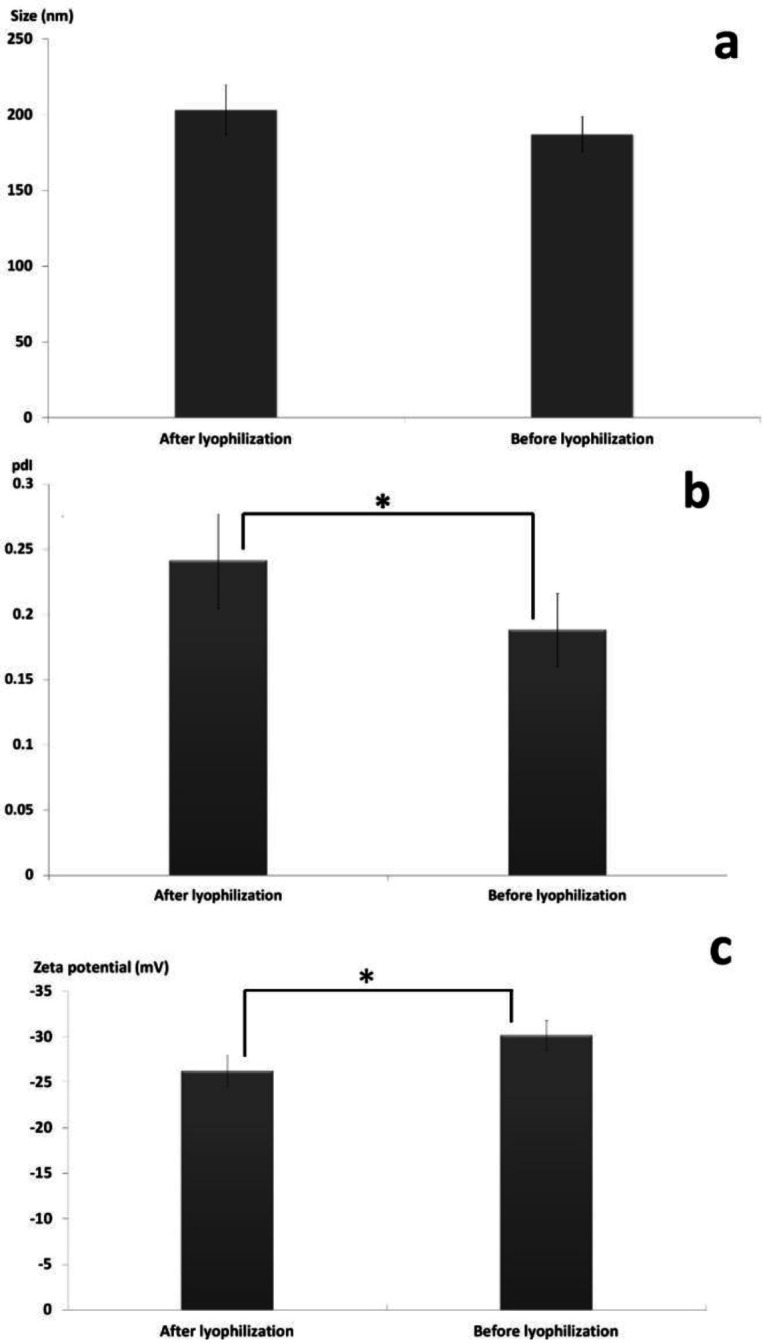
Influence of lyophilization on SLNs characteristics, (a) particle size (nm) (b) pdI (c) zeta potential (mV). Data represent Mean ± SD, n = 5. *Results are significantly different, *p* < 0.05.

The effect of freeze drying process in the presence of the cryoprotectant (*i.e.* sucrose 5% w/v) on physicochemical characteristics of the SLNs including particle size, PdI and zeta potential was investigated and the appropriate results are illustrated in Figure 4. Previous studies revealed that di-saccharides such as sucrose are more efficient cryoprotectant compared to mono-saccharides and consequently exhibit higher efficiency in conserving the physicochemical features of nanoparticles during lyophilization (40, 41).

As shown in Figure 4a, although the size of the SLNs was slightly increased from 187 ± 11.97 nm to 203 ± 16.49 nm during lyophilization, statistical analysis of data using two independent sample t-test revealed no significant differences in the size of the nanoparticles before and after freeze-drying (*p*-value > 0.05). Figure 4b revealed that the PdI of the nanoparticles was significantly increased from 0.188 ± 0.028 to 0.241 ± 0.036 during the lyophilization (*p*-value < 0.05). Determination of zeta potential is an effective method to consider the eventual interactions between the cryoprotectant molecules and the nanoparticles surface (42, 43). It was showed that the zeta potential of the particles was significantly decreased from -30.16 ± 1.6 mV to -26.24 ± 1.71 mV (*p-*value < 0.05) due to accumulation of the sucrose molecules at the surface of the nanoparticles which can lead to establishment of hydrogen bonds and consequently masking the negative charges of the lipids (Figure 4c) (44, 45).

*Preparation and Characterization of DPI*

In this study, lyophilization technique was employed to prepare the DPI formulations. For this purpose, various concentrations of lactose (*i.e.* 1, 5, 10, 15 and 20%w/v) as the inhalational carrier were applied to provide the intended dispersions. Each formulation was freeze dried over a period of 72 h and analyzed by Anderson cascade impactor. 

As can be observed in Table 6, analysis of the results showed that by increasing the lactose concentration in the SLNs dispersions, the particle size of the DPIs was increased. Pisponen *et al. *suggested that the observed particle size increasing of DPI formulations followed by increase in lactose concentration can be due to classical nucleation theory (46). Lactose is one of the major factors initiating the nucleation process in a solution. Accordingly, in a more concentrated solution of lactose nuclear growth can be promoted and more lactose molecules crystallized.

 DPIs with particle size values above 5µm and below 1 µm cannot access the peripheral airways properly. This is due to their immediate elimination after deposition in oral cavity (*i.e.* > 5 µm) and their little and slow deposition which is affected by Brownian motion (*i.e.* < 1 µm) (47). Therefore, the optimal size for pulmonary drug delivery of the particles is in the range of 1µm to 5 µm in aerodynamic diameter. Fine particle fraction (FPF) is defined as the percentage of particles with aerodynamic diameter between 1 µm to 5 µm which can access to the alveoli. As indicated in Table 6, the calculated FPFs were 35.71 ± 1.81%, 53.96 ± 3.67%, 72.57 ± 4.33%, 54.99 ± 3.04%, and 22.03 ± 2.53% in various DPI formulations containing lactose 1%, 5%, 10%, 15% and 20% w/v, respectively. It was revealed that the highest FPF% was obtained using lactose 10% and therefore, this formulation was suggested to be efficient for drug delivery to the peripheral airways. 

*In-vitro release study*

**Figure 5:**
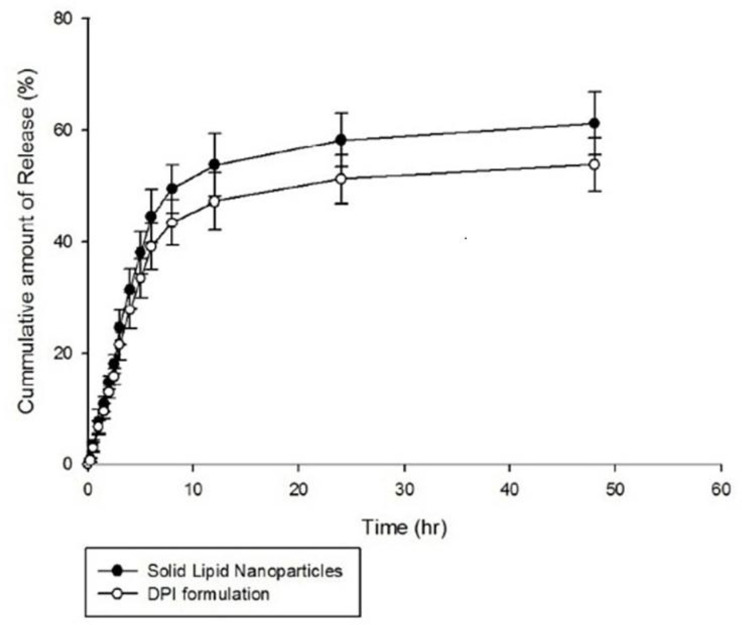
Cumulative AMB release profile from the SLNs and DPI formulation (PBS, pH 7.4, n = 3).

The *in-vitro *release of AMB from the optimized SLNs and also DPI formulations was evaluated in phosphate buffer saline (PBS) adjusted to a pH value of 7.4. The results are illustrated in Figure 5. As shown in the figure, 58.23 ± 4.89% and 61.22 ± 5.70% of entrapped AMB was released from the optimized SLNs over a period of 24 h and 48 h, respectively indicating a slow and sustained release behavior. *In-vitro *release of AMB from the dry powders was also determined using formulation of 10 %w/v of lactose and the similar sustained release behavior was obtained. Similarly, various studies reported slow and sustained release behavior of drugs encapsulated into SLNs preparations (48-50). Therefore, SLNs are suggested as suitable carriers for prolonged and sustained drug release (36) and this can be achieved when drug is homogenously dispersed into the lipid matrix and can only be released through diffusion (47). Moreover, in the study performed by Kushwaha *et al.*, it was suggested that the slow release of drug from SLNs may be due to increased diffusional distance and hindrance effects of lipid shells which prevent surrounding aqueous medium to penetrate inside the particles and release the encapsulated through dissolution mechanism (50). 

The drug release data were fitted to various mathematical kinetic models including zero order, first order, Higuchi, Hixon–Crowell and Korsmeyer-Pepas using Sigma-plot® software (version 10.0.0.54). As shown in Table 7, release kinetic of the both optimized SLNs formulation and DPI preparation were best fitted to the first order kinetic model. In this study, the sustained release of AMB from the optimized SLNs describes the diffusion of the drug from homogenous matrix system which is in well accordance with fickian diffusion mechanism explained by the first order release kinetic model. The studies performed by Priyanka and Hasan revealed first order release kinetic of all montelukast-loaded SLNs formulations (51). Moreover, the study of Kakkar *et al.* showed first order release kinetic of curcumin from SLNs (52). 

*Morphology of the particles*

**Figure 6:**
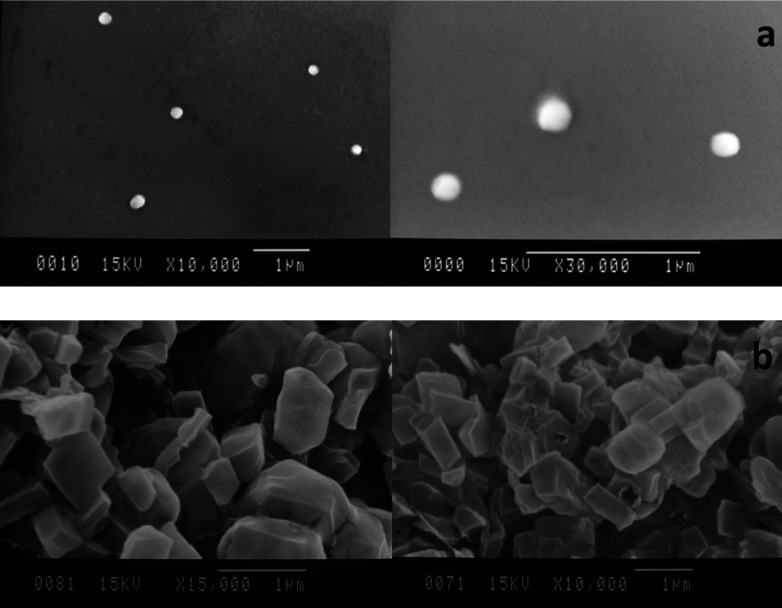
SEM images of the particles, (a) optimized SLNs; (b) DPI formulation

Scanning electron micrographs of the SLNs and DPI formulations are illustrated in Figure 6. As shown in Figure 6a, the SEM images revealed a spherical shape and smooth surface particles with diameters in accordance with data obtained by photon correlation spectroscopy (PCS). The DPI samples using formulation of 10% w/v of lactose were also examined and the SEM images showed a cubic shape and smooth surface particles (Figure 6b). No sign of aggregation was detected in the SEM images.

**Table 1:** Ranges and constrains of variables used in experimental design

**Independent variables (factors)**	**Levels**	
	-1	+1
A: GMS/Lecithin ratio	0.25	3
B: PEG 400 (w/v%)	0	3
C: Tween 80 (w/v%)	0.25	4
D: Emulsifying time (h)	0.25	3
E: Cooling time (h)	0.25	2
Dependent variables (responses)	Constrains	
Y_1 _= size (nm)	Minimize	
Y_2 _= pdI	Minimize	

**Table 2:** Fractional factorial experimental design (n = 3).

** Dependent variables (responses)**		**Independent variables (factors)**					**Formulation no.**
**Y**_2_ **(mean ± SD)**	**Y**_1_ ** (nm)** **(mean ± SD)**	**E**	**D**	**C**	**B**	**A**	
0.30 ± 0.01	178.20 ± 6.70	2.00	0.25	0.25	3.00	0.25	F_1_
0.22 ± 0.04	203.00 ± 8.40	0.25	0.25	0.25	0.00	3.00	F_2_
0.22 ± 0.01	190.20 ± 4.80	0.25	3.00	0.25	3.00	3.00	F_3_
0.24 ± 0.01	181.90 ± 5.30	1.13	1.63	2.13	1.50	1.63	F_4_
0.49 ± 0.03	176.70 ± 5.09	2.00	3.00	0.25	0.00	0.25	F_5_
0.17 ± 0.01	178.30 ± 4.10	2.00	3.00	4.00	3.00	3.00	F_6_
0.25 ± 0.02	190.00 ± 2.82	1.13	1.63	2.13	1.50	1.63	F_7_
0.24 ± 0.03	201.00 ± 4.24	1.13	1.63	2.13	1.50	1.63	F_8_
0.21 ± 0.02	179.60 ± 12.44	0.25	3.00	4.00	0.00	0.25	F_9_
0.42 ± 0.10	256.00 ± 60.81	0.25	0.25	4.00	3.00	0.25	F_10_
0.37 ± 0.02	393.60 ± 47.50	2.00	0.25	4.00	0.00	3.00	F_11_

**Table 3:** Characteristics of the best fitted mathematical models.

**Adeq Precision**	**adjusted R-Squared**	**R-Squared**	**CV**	**Best fitted model**	**Response factor**
25.0130	0.9792	0.9954	4.5300	2FI	Particle size
64.4350	0.9970	0.9993	1.9200	2FI	pdI

**Table 4.** Optimized independent variables and predicted responses

Desirability	Predicted dependent variables (responses)	Optimized Independent Variables					
0.86	Y_2_ = PdI	Y_1_ = size (nm)	E: Cooling time (h)	D: Emulsification time (h)	C: Tween 80 (w/v%)	B: PEG 400 (w/v%)	A: GMS/ Lecithin
	0.172	174.63	0.27	2.78	2.11	0.15	2.46

**Table 5:** The observed responses for predicted optimized formulations (n = 5).

**Optimized SLNs characteristics**			**Dependent variable (responses)**			
**DL (%)**	**EE (%)**	**Zeta (mV)**	**pdI**		**Size (nm)**	
Observed response (Mean ± SD**)**	Observed response (Mean ± SD)	Observed response (Mean ± SD)	Prediction Error (%)	Observed response (Mean ± SD)	Prediction Error (%)	Observed response (Mean ± SD)
2.76 ± 0.32	89.30 ± 3.47	-30.16 ± 1.60	+ 8.51	0.188 ± 0.028	+ 6.63	187.04 ± 11.97

**Table 6:** Aerodynamic Size distribution of different formulations of the DPIs using Andersoncascade impactor (n = 3)

**Fine Particle Fraction (FPF) (%) Mean ± SD**	**Drug deposition (%)**								**Lactose (%)**	**No.**
	**Stage 8 (0.52 µm) Mean ± SD**	**Stage 7 (0.93 µm) Mean ± SD**	**Stage 6 (1.55 µm) Mean ± SD**	**Stage 5 (3.5 µm) Mean ± SD**	**Stage 4 (5.0 µm) Mean ± SD**	**Stage 3 (9.8 µm) Mean ± SD**	**Stage 2 (14.8 µm) Mean ± SD**	**Stage 1 (21.3 µm) Mean ± SD**		
35.71 ± 1.81	32.54 ± 3.62	26.50 ± 3.42	6.48 ± 1.31	2.73 ± 0.62	2.44 ± 0.86	0.67 ± 0.12	ND^1^	ND^1^	1	1
53.96 ± 3.67	25.87 ± 4.72	24.61 ± 2.65	17.93 ± 3.42	11.42 ± 3.15	9.27 ± 2.38	4.68 ± 1.47	1.31 ± 0.18	ND^1^	5	2
72.57 ± 4.33	10.31 ± 3.57	23.52 ± 4.11	27.10 ± 3.95	21.95 ± 4.83	4.39 ± 2.52	5.61 ± 1.83	2.24 ± 0.68	2.93 ± 0.73	10	3
54.99 ± 3.04	2.63 ± 0.96	10.88 ± 2.75	18.43 ± 3.52	25.68 ± 2.41	11.46 ± 3.78	17.13 ± 3.27	7.49 ± 1.59	8.74 ± 2.86	15	4
22.03 ± 2.53	ND^1^	4.94 ± 1.21	7.37 ± 3.64	9.72 ± 2.31	15.53 ± 3.64	18.90 ± 2.75	22.83 ± 3.56	20.37 ± 3.63	20	5

^1^Not Detected.

**Table 7:** Parameters of the drug release kinetics

**Fitted Theoretical Models**	** R** ^2^		**Adjusted R** ^2^		** constants**	
	**SLNs**	**DPIs**	**SLNs**	**DPIs**	**SLNs**	**DPIs**
Zero Order	0.0000	0.0000	0.0000	0.0000	_	_
First order	0.9862	0.9861	0.9839	0.9838	K_1 _= 0.176	K_1 _= 0.176
Hixon-Crowell	0.0000	0.0000	0.0000	0.0000	_	_
Higuchi	0.7925	0.7922	0.7765	0.7762	K_h _= 0.1951	K_h _= 0.1952
Korsmeyer-Pepas	0.8466	0.8464	0.8348	0.8346	K_p _= 0.269 n = 0.385	K_p _= 0.269 n = 0.385

## Conclusion

This study focuses on the preparation and *in-vitro *characterization of a DPI formulation containing SLNs encapsulating AMB. The effects of the formulation variables including concentration ratio of GMS/lecithin, concentration of tween 80 and PEG 400 along with emulsification time and cooling time on physicochemical properties of the nanoparticles were studied based on fractional factorial experiments. The optimized nanoparticles were characterized as the smallest in size and lowest in PdI. Morphological study of the SLNs revealed formation of non-aggregated, uniformly sized and spherical shape particles with smooth surfaces. Lyophilization technique was employed successfully to stabilize the nanoparticles and preparation of the dry powder inhalers accompanying with lactose as the inhalational carrier. It was revealed that the highest FPF% was obtained using 10% w/v of lactose and therefore, this formulation was suggested to be efficient for drug delivery to the peripheral airways. Morphological study of the DPI formulations showed formation of non-aggregated, uniformly sized particles with smooth surfaces. *In-vitro *release studies were performed on the both nanosuspensions and DPI preparations and the results showed sustained release profile of AMB over a period of 48 h and the kinetic of release was best fitted to the first order kinetic model. 
